# Pre-hospital transfusion of packed red blood cells in 147 patients from a UK helicopter emergency medical service

**DOI:** 10.1186/s13049-017-0356-2

**Published:** 2017-02-14

**Authors:** Richard M. Lyon, Eleanor de Sausmarez, Emily McWhirter, Gary Wareham, Magnus Nelson, Ashley Matthies, Anthony Hudson, Leigh Curtis, Malcolm Q. Russell, on behalf of Kent, Surrey & Sussex Air Ambulance Trust

**Affiliations:** 1 Kent, Surrey & Sussex Air Ambulance Trust, Wheelbarrow Park Estate, Pattenden Lane, Marden, Kent, TN12 9QJ UK; 2 Department of Emergency Medicine, St George’s University Hospitals NHS Trust, London, UK; 3 University of Surrey, Surrey, UK

**Keywords:** Haemorrhage, Pre-hospital care, Trauma

## Abstract

**Background:**

Early transfusion of packed red blood cells (PRBC) has been associated with improved survival in patients with haemorrhagic shock. This study aims to describe the characteristics of patients receiving pre-hospital blood transfusion and evaluate their subsequent need for in-hospital transfusion and surgery.

**Methods:**

The decision to administer a pre-hospital PRBC transfusion was based on clinical judgment. All patients transfused pre-hospital PRBC between February 2013 and December 2014 were included. Pre-hospital and in-hospital records were retrospectively reviewed.

**Results:**

One hundred forty-seven patients were included. 142 patients had traumatic injuries and 5 patients had haemorrhagic shock from a medical origin. Median Injury Severity Score was 30. 90% of patients receiving PRBC had an ISS of >15. Patients received a mean of 2.4(±1.1) units of PRBC in the pre-hospital phase. Median time from initial emergency call to hospital arrival was 114 min (IQR 103–140). There was significant improvement in systolic (*p* < 0.001), diastolic (*p* < 0.001) and mean arterial pressures (*p* < 0.001) with PRBC transfusion but there was no difference in HR (*p* = 0.961). Patients received PRBC significantly faster in the field than waiting until hospital arrival. At the receiving hospital 57% required an urgent surgical or interventional radiology procedure. At hospital arrival, patients had a mean lactate of 5.4(±4.4) mmol/L, pH of 6.9(±1.3) and base deficit of −8.1(±6.7). Mean initial serum adjusted calcium was 2.26(±0.29) mmol/L. 89% received further blood products in hospital. No transfusion complications or significant incidents occurred and 100% traceability was achieved.

**Discussion:**

Pre-hospital transfusion of packed red cells has the potential to improvde outcome for trauma patients with major haemorrhage. The pre-hospital time for trauma patients can be several hours, suggesting transfusion needs to start in the pre-hospital phase. Hospital transfusion research suggests a 1:1 ratio of packed red blood cells to plasma improves outcome and further research into pre-hospital adoption of this strategy is needed.

**Conclusion:**

Pre-hospital PRBC transfusion significantly reduces the time to transfusion for major trauma patients with suspected major haemorrhage. The majority of patients receiving pre-hospital PRBC were severely injured and required further transfusion in hospital. Further research is warranted to determine which patients are most likely to have outcome benefit from pre-hospital blood products and what triggers should be used for pre-hospital transfusion.

## Background

Haemorrhage is one of the leading causes of death in major trauma patients [1]. Major haemorrhage is defined as bleeding in excess of 150 mL/min or 50% of the patient’s total blood volume in less than 3 h [2]. Cardiac arrest as a result of major haemorrhage contributes to a significant proportion of pre-hospital trauma deaths and has been shown to have a poor outcome [1]. Uncontrolled haemorrhage may result in the development of the lethal triad of hypothermia, coagulopathy and acidosis. Each of these physiological disturbances contributes to the development of acute traumatic coagulopathy. Hypoperfusion leads to cellular hypoxia, leading to anaerobic metabolism and a metabolic acidosis. Anaerobic metabolism will also limit endogenous heat production exacerbating any hypothermia, which will worsen coagulopathy [3]. This worsening cycle can rapidly result in death unless haemorrhage is stopped, resuscitation commenced and the coagulopathic state reversed [4].

The management of hypotensive trauma patients has changed significantly in recent years, particularly with new research and innovation from the military conflict zones. New strategies include minimal crystalloid resuscitation and earlier blood product transfusion with rapid transfer for damage control resuscitation [5]. Historically, crystalloids have commonly been administered to trauma patients with suspected bleeding in the pre-hospital phase. However, the administration of crystalloid fluids alone can result in dilutional anaemia, dilutional coagulopathy and reduced blood oxygen carrying capacity [6]. The use of Packed Red Blood Cells (PRBC) provides more effective volume expansion and increased oxygen carrying capacity [7] and is therefore routinely used during hospital resuscitation of the major trauma patient, with major trauma centres storing PRBC for immediate administration. For patients in traumatic cardiac arrest, administration of PRBC is more likely to achieve a return of spontaneous circulation than a crystalloid or colloid infusion [8].

Early transfusion of PRBC in patients with major haemorrhage following trauma has been shown to improve outcome [7]. Depending on geography, major trauma victims must often be transported significant distances to hospital. If PRBC are to be administered early, it is likely they will need to be given in the pre-hospital phase in these patients. Helicopter Emergency Medical Service (HEMS) teams have begun to transfuse PRBC with initial research showing favourable outcomes [9–13]. Brown et al. [13] matched patients receiving early PRBC transfusion to controls and showed an increased 24-h survival, lower odds ratio of shock and decreased 24-h PRBC requirement. In patients presenting in cardiac arrest following trauma, without the option of pre-hospital PRBC transfusion, Lockey et al. [1] reported that 7.5% survived to hospital discharge and Willis et al. reported a 5% survival rate to discharge [14]. More recently Weaver et al. [9] reported a return a spontaneous circulation rate of 45% in patients who suffered traumatic cardiac arrest on scene and received PRBC, inferring that PRBC may be responsible for the improved resuscitation in this cohort.

There are several barriers to pre-hospital blood product transfusion. These include expense, risk of transfusion reactions and disease transmission, short shelf-life and concerns over wastage, problematic storage and the need to achieve complete traceability [12].

A potential complication associated with PRBC transfusion is hypocalcaemia. The influence of PRBC on serum calcium levels is largely due to the addition of citrate. Recipient citrate levels can accumulate in massive transfusion [15]. Increased plasma citrate leads to chelation of calcium ions. Hypocalcaemia is potentially detrimental to the trauma patent as calcium regulation is critical for normal cell function, neural transmission, membrane stability, bone structure, blood coagulation and intracellular signalling [16]. It is associated with increased mortality from increased risk of arrhythmia and a decrease in cardiac contractility [15]. Calcium is a potent inotrope and severe cardiac depression and hypotension can result from low levels [17].

The UK military currently recommends that ionised calcium levels should be kept above 1.0 mmol/L by the administration of 10 mL of 10% calcium chloride with each “shock pack” (specified pre-packed blood products for rapid issue: initially 2 units PRBC and 2 units Fresh Frozen Plasma (FFP)) [18].

Studies to evaluate the outcome of major trauma patients who receive pre-hospital PRBC versus crystalloid are on-going and the optimum triggers to initiate pre-hospital transfusion remain unknown.

Understanding which patients have an on-going transfusion requirement upon hospital arrival, will help inform pre-hospital transfusion practice, in particularly guiding transfusion triggers.

The aim of this study is to describe the characteristics, physiology, clinical interventions and outcomes of civilian trauma patients receiving pre-hospital PRBC from a UK Helicopter Emergency Medical Service (HEMS).

## Methods

### Setting

Kent, Surrey & Sussex Air Ambulance Trust (KSSAAT) provides two doctor-paramedic teams, one of which operates 24-h a day and the other 12-h a day. The teams can deploy by helicopter or response car. The KSSAAT HEMS service provides enhanced pre-hospital medical care to south east England, a static population of approximately 4.3 million and a transient population of up to a total of 10 million. This area consists of both urban and rural areas and is served by Major Trauma Centres in London, Brighton and Southampton [19]. Patient transport to hospital can be by air or road, depending on geography, weather, time of day and hospital helipad availability. On average, patients are 44 km in linear distance from incident scene to receiving hospital and have an average primary transfer time of 30 min to hospital by air or road. The maximum road transfer time is approximately 1 h 45 min from the furthest geographical point of the region. Statistics from The National Audit Office suggest that in this region of the UK, there are approximately 630 cases of major trauma annually [20].

In February 2013, KSS HEMS started carrying four units of PRBC, based on current hospital and military guidance and a pragmatic view of the published literature. PRBC are provided by two nearby hospital transfusion laboratories and procedures are in place to ensure full traceability of units and compliance with the Blood Safety and Quality Regulations (2005) and Medicines and Healthcare Products Regulatory Agency [21].

The decision on-scene to administer blood is made by the HEMS team in accordance with the relevant KSS HEMS Standard Operating Procedure (SOP). The SOP states that a pre-hospital blood transfusion is indicated for patients showing signs of severe haemorrhagic shock following trauma. The clinical assessment of this is based on history, mechanism of injury, patient physiology and response to resuscitative efforts (e.g. lack of response to fluid given by other pre-hospital providers). Permissive hypotension is practised and currently ventilated patients receive blood products to achieve a radial pulse/blood pressure 80 mmHg only. In patients with an isolated head injury, the target is for a systolic above 100 mmHg. A key trigger for initiating blood transfusion in the ventilated patient is therefore loss of radial pulse. In the awake blunt trauma patient, loss of verbal contact is used as a trigger. In a patient with penetrating torso trauma, packed cells are used to maintain a central (carotid) pulse only.

In addition, the SOP states that tranexamic acid should be given if PRBC are transfused, and that PRBC should be transfused warm, via a filtered blood administration set, at an appropriate rate based on patient physiology. KSSAAT currently use the Belmont Buddy Lite system to administer warmed blood products. All patients should be ‘packaged’ with bubble-wrap and a warming mat regardless of time of year, to minimise heat loss and hypothermia.

### Study subjects

All patients who received pre-hospital PRBC from KSS HEMS between 1^st^ February 2013 and 10^th^ December 2014 (677 days) were included in the data set as a convenience sample. A HEMS paramedic in the ambulance emergency operations control room screened all incoming calls to identify missions appropriate for HEMS. In addition, local ambulance crews could request HEMS’ attendance. In the 2014 calendar year, KSS HEMS attended 1712 incidents [22]. Patient data for all HEMS missions are routinely entered onto a specific HEMS database.

### Data collection

Patient demographic and clinical data were collected for all patients who received blood products. Data obtained from HEMSbase was analysed retrospectively. Data fields analysed included sex, age, mechanism of injury, scene distances and times, HEMS’ arrival time, physiology, HEMS’ interventions, reason for transfusion, pre-transfusion physiology, number of units of PRBC and pre-hospital response to PRBCs.

In-hospital data and outcomes were obtained retrospectively from in-hospital records. The data fields analysed included physiology on arrival, initial blood gas results, blood products transfused in the Emergency Department, blood products transfused in the first 24 h, surgical and interventional radiology procedures, post-Emergency Department destination, 6 h mortality, 28 day mortality, Intensive Treatment Unit length of stay and hospital length of stay. Massive transfusion was defined as either 5 units PRBCs in the Emergency Department or 10 units PRBCs in 24 h [23] (including those units given by HEMS). The Injury Severity Score (ISS) score at hospital arrival was recorded.

### Statistical analysis

Patient characteristics and outcomes are reported using descriptive statistics with mean (with SDs) and median (with IQR). Parametric data were analysed using unpaired t-tests or ANOVAs with Bonferroni post-hoc testing. Shapiro-Wilk and Kolmogorov-Smirnov tests were performed on all parametric data to check for normality. The level of significance was set at p ≤ 0.05. Data was entered into Microsoft Office Excel Spreadsheet (Microsoft Corporation, Version 7). All other data analysis performed on SPSS (IBM, Version 22) for Windows.

### Ethical approval

This study met UK National Institute for Health Research criteria for a service evaluation. All the data utilised for this study was routinely collected as part of standard pre-hospital and hospital patient data collection. Formal ethical approval was therefore not required as criteria for service evaluation were met. The study was registered with St George’s NHS Trust and the University of Surrey as a service evaluation.

## Results

One hundred forty-seven patients were transfused PRBC during the study period. 114 patients were male (78%). Median age was 42 years (range 9–92 years).

Pre-hospital data were obtained for all 147 patients, but of the 109 (74%) patients taken to hospital, records were only available for 85 (78%) of these. 38 (26%) patients were declared deceased on scene.

All patients were thought to be suffering with traumatic haemorrhagic shock except for 5 (3%). These were made up of 2 gastrointestinal bleeds, 2 suspected ruptured abdominal aortic aneurysms and 1 ruptured abscess/pseudoaneurysm in an injecting drug user. One of these patients was declared deceased on scene.

**Fig. 1:**
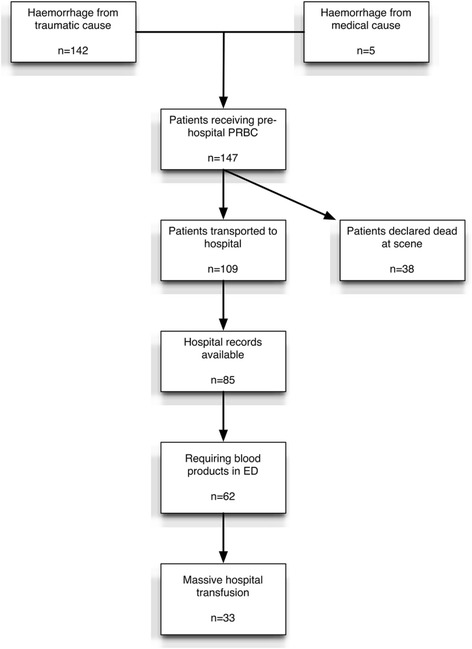
Inclusion of patients

Of the 142 traumatic injuries, 128 were of blunt mechanism of injury (90%) and 103 (73%) were involved in Road Traffic Collisions (RTC). Patients had a median ISS of 33 and 90% had an ISS of greater than 15. A summary of injury mechanism is given in Table 1. A summary of included patients is shown in Fig. 1.

**Table 1:** Mechanism of injury. Note 1 patient did not have a mechanism of injury documented

Mechanism of Injury	Number	%
RTC Car Passenger/Driver	42	28.6
RTC Pedestrian	29	19.7
RTC Motorcyclist	26	17.7
Fall from Height	17	11.6
Stabbing (Central or Peripheral)	9	6.1
RTC Cyclist	6	4.1
Hit by Train	5	3.4
Spontaneous, non-traumatic bleed	5	3.4
Machinery/Crush injury	3	2.0
Shooting	2	1.4
Assaulted	1	0.7
Burns and abdominal stabbing	1	0.7
Fall from standing	1	0.7

### Pre-hospital cardiac arrest

On HEMS arrival, 103 (70%) of patients had spontaneous circulation. Circulation was lost in 16 (16%) patients, but return of spontaneous circulation was achieved in 10 (63%) of these. 5 (31%) of these patients were subsequently declared deceased on scene. One patient never regained spontaneous circulation but was taken to the Emergency Department where they were declared deceased. Of the 44 (30%) of patients without spontaneous circulation on HEMS arrival, return of spontaneous circulation was achieved in 6 (14%). 33 (75%) were declared deceased on scene and the remaining 5 (11%) never regained circulation but were taken to hospital and were then declared deceased in the Emergency Department.

**Fig. 2:**
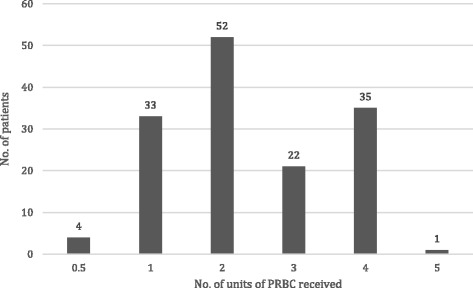
Frequency distribution of number of units of PRBC transfused pre-hospital. One patient received 5 units of PRBC as two HEMS were at the same incident, meaning more PRBC were available

Patients received a mean (SD) 2.4 (±1.1) units of PRBC. The frequency distribution of transfused PRBC is shown in Fig. 2. A single patient received 5 units as two HEMS teams were present at the same incident, so two PRBC supplies were available.

Of the 147 patients to receive pre-hospital PRBC, 56 (38%) patients had a transfusion initiated because they had no palpable central pulse. The other reasons cited for starting transfusion included a measured drop in non-invasive blood pressure (39.9%), loss of peripheral pulse (9.1%), tachycardia (2.0%) and a loss of verbal contact (1.4%). For 9.6% patients it was a combination of these reasons.

Other pre-hospital interventions in this group included RSI (50%), thoracostomies (66%) and resuscitative thoracotomy (4%).

### Transport to hospital

One hundred nine (74%) patients were taken to hospital. Median straight line distance from scene to the receiving hospital was 43.8 km by air (IQR 27.6 – 72.4) and 55.4 km by road (IQR 36.9 – 84.7). Median time from ‘999’ call to hospital arrival was 114 min (IQR 103–140 min). Median (IQR) time for HEMS team to reach the patient after initial ‘999’ call was 34.5 (25.0 – 48.8) minutes. Patients were therefore able to receive PRBC significantly faster than had pre-hospital PRBC not been available. A summary of the demographics and clinical interventions is shown in Table 2.

**Table 2:** Summary of patients receiving pre-hospital PRBC

No. of patients administered PRBCs	147
Male sex *n* (%)	114 (78%)
Mean age years (range)	42.3 (9–92 years)
Penetrating injury *n* (%)	14 (10%)
Blunt injury *n* (%)	128 (87%)
Non-injury *n* (%)	5 (3%)
RTC related *n* (%)	103 (73%)
Mean units PRBCs administered (SD)	2.4 (1.1)
Received RSI *n* (%)	73 (50%)
Thoracostomy *n* (%)	97 (66%)
Thoracotomy *n* (%)	6 (4%)
Median distance to major trauma centre (IQR) in km	43. 8 (27.6 – 72.4)
Median (IQR) time from 999 call to HEMS arrival in mins	34.5 (25.0 – 48.8)
Median (IQR) HEMS scene time in mins	46.0 (34.5 – 63.0)
Median (IQR) time from HEMS arrival to receiving hospital in mins	76.0 (66.0 – 98.0)
Median (IQR) time from ‘999’ call to arrival at receiving hospital in mins	114.0 (103.0 – 140.0)

### Hospital care

Of the 109 patients taken to hospital, full records were available for 75 (68%).

### Pre-hospital patient physiology

**Fig. 3:**
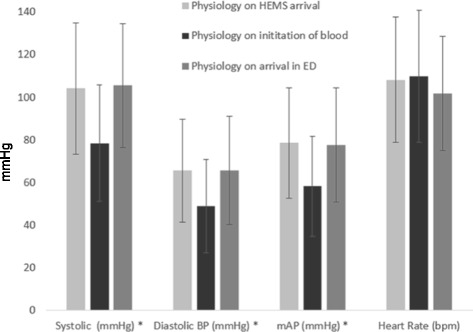
Mean (SD) physiological parameters on arrival of HEMS team, initiation of blood and arrival in the ED. * Denotes significant difference (*p* < 0.05) between value on initiation of blood when compared to arrival in the ED

Figure 3 shows the difference between physiology at the time of HEMS’ arrival; initiation of PRBC transfusion; and arrival at the Emergency Department. Patients transfused with PRBC showed significant increase in systolic (p < 0.001), diastolic (p < 0.001) and mean arterial blood pressures (p < 0.001) between the time immediately before transfusion and the time of arrival at the Emergency Department. There was no difference in HR (p = 0.96).

### At hospital

Of the blood gases taken on arrival at hospital, 50% were arterial in nature and 50% venous, for which the mean values are shown in Table 3.

**Table 3:** Arrival venous blood gas values on arrival at hospital

	Minimum	Maximum	Mean	Std. Deviation
pH	6.60	7.42	7.15	0.17
Base Deficit (mEq/L)	–28.20	0.40	–9.48	6.82
Lactate (mmol/L)	0.90	19.90	5.27	4.08

### In-hospital transfusion

Further blood product data was available for 70 (82.3%) of those patients taken to hospital. Of these, 62 (89%) went on to have further blood products and 33 (47%) went on to have a massive transfusion. 7 patients (4% of those transfused pre-hospital) did not receive any blood products in hospital.

### In-hospital intervention

For the patients whose hospital notes were available (*n* = 75), 27 (36%) had surgery and 6 (8%) patients had an interventional radiology procedure directly after moving from the Emergency Department.

Operations were most commonly laparotomies (most commonly for splenectomies); vascular operations for repair to damaged vessels and lone bone fracture fixations. Other procedures included thoracotomy, pelvic fixation, craniotomy and fasciotomy. Some patients had more than one type of surgery. Radiological interventions included embolisation.

The immediate post-Emergency Department destination was Intensive Care for 51%, operating theatre for 29% and the trauma ward for 6%.

### Patient outcome

Eleven patients (15%) were declared deceased in the Emergency Department. For those admitted alive (*n* = 64), median Intensive Care length of stay was 6 days (IQR 2–17) and hospital stay was 18 days (IQR 3–32). 21 patients were transferred to other hospitals. This was for patients returning to the local hospitals in 57% (including 1 international transfer) and other specialist input in the others. For those who survived to hospital, survival at 6 h post-arrival was 84% and at 28 days was 70%.

The patients in this study had a mean ionised calcium of 1.1 mmol/L on arrival in the Emergency Department. Blood gas calcium was significantly lower with increasing volume of PRBCs transfused (*p* = 0.03).

100% traceability of pre-hospital PRBC was achieved. There were no protocol violations. Eight units of PRBC were wasted soon after introduction of the transfusion protocol due to user error. No immediate transfusion complications or high-risk incidents occurred.

## Discussion

The majority of patients attended by the HEMS team in this cohort were severely injured with a mean (SD) ISS of 32.9 (±13.41) and 90% of patients had an ISS of >15. Administering PRBC in the pre-hospital setting was able to significantly reduce the time to first transfusion. Without a system of administering pre-hospital blood products, the majority of these severely injured patients would have a delay of almost 2-h to receive blood products in hospital. A delay of more than an hour to administer PRBC for a seriously injured patient with cardiovascular compromise is likely to adversely affect coagulopathy and outcome. The patients in this cohort showed significant signs of haemorrhagic shock with high levels of serum acidosis, deranged base excess and raised lactate. Given the frequency of transfusion was, on average, every 4.6 days, and the acuity of the patients was so high, it is likely that PRBC transfusion in the pre-hospital phase does have a role in the management of acutely haemorrhagic patients. Moving forward, recent studies suggest that a 1:1 ratio of PRBC to plasma is likely to be more beneficial when resuscitating patients with haemorrhagic shock. At the time of this evaluation, lyophilised plasma was not available in the UK. KSSAAT are currently evaluating use of lyophilised plasma in the field.

In this study, the majority of patients had a blunt injury with the most common mechanism being involvement in a road traffic collision. This is similar to the injury distribution reported by Sherren et al. [10] for Sydney HEMS.

Patients with spontaneous circulation on HEMS’ arrival who required PRBC showed less physiological shock on arrival at hospital, as evidenced by blood pressure on admission, when compared to pre-hospital observations. This echoes the findings by Brown et al. [13] who found that pre-hospital PRBC transfusion was associated with lower odds of shock and suggests that pre-hospital transfusion is of value in preventing tissue hypoperfusion.

We found that 89% of patients went on to receive further blood products in the ED, yet only 44% required damage control surgery. This may be that patients received adequate initial resuscitation but further research is required, particularly to guide triggers for transfusion in the pre-hospital and ED settings.

In this study, the group of hypovolaemic traumatic cardiac arrest or peri-arrest patients had poor outcomes as previously described by the literature. However, a ROSC rate of 14% in patients in TCA on HEMS’ arrival and 63% for those who suffered a traumatic cardiac arrest in HEMS’ presence is above that expected and previously documented [9, 24]. Patients in traumatic cardiac arrest following hypovolaemia are unlikely to gain return of spontaneous circulation with a crystalloid infusion and this high return of spontaneous rate may be attributable to blood product use. Further research is required into the pre-hospital management of traumatic cardiac arrest to define which of patients are most likely to benefit from PRBC transfusion.

Early activation of massive transfusion protocols ensures the provision of appropriate blood products quickly. These protocols have been associated with improved survival [25] through the treatment of intrinsic acute traumatic coagulopathy and prevention the development of dilutional coagulopathy [26]. Furthermore, the early recognition of massive haemorrhage should decrease the time to damage control surgery and definitive treatment. Dailey [27] describes one system for activating a massive transfusion protocol (a patient being declared “Code Red”) based on patient physiology. ‘Code Red’ was triggered when a patient had a systolic blood pressure (BP) of less than 90 mmHg with a poor response to initial fluid resuscitation in the context of suspected active haemorrhage. Several scoring systems have been developed to aid the early recognition of acute exsanguination. Brockamp et al. [25] examined 6 scoring systems and found that those with weighted and the greatest number of parameters performed better. However, many of the parameters, such as haemoglobin, base excess and lactate are not currently measured in the pre-hospital phase, so for our HEMS teams, the decision to transfuse is based on clinical findings and judgment. Given that 89% of patients given PRBCs in the pre-hospital phase went on to have further blood products, 44% had an immediate haemorrhage control procedure and 48% met massive haemorrhage definition, we suggest that HEMS teams can reasonably predict the need for massive transfusion based on clinical judgment alone, with loss of radial pulse in a ventilated patient being a useful objective indicator. The addition of near patient testing, may improve accuracy and further research is warranted in this field.

Blood gas ionised calcium was significantly lower with increasing units of transfused PRBCs transfused; an expected relationship due to the addition of citrate into blood products [2], and fluid resuscitation causing haemodilution of the remaining ions [28, 29]. Treatment of hypocalcaemia during transfusion is generally recommended for patients with an ionised calcium below 1.0 mmol/L [15]. The patients in this study had a mean ionised calcium of 1.1 (which is just below the lower limit of normal). Therefore, on average, patients were just above the treatment threshold. However, administration of calcium is likely to improve cardiovascular function and should therefore be considered – either in the pre-hospital phase or early in-hospital.

This study demonstrates that PRBC transfusion in the pre-hospital phase is both feasible and safe for a UK physician-led, civilian HEMS service underpinned by a rigorous clinical governance system. Compliance with all NHS documentation and legislation is possible by strict adherence with local SOPs developed in conjunction with local NHS blood product providers.

This study has several limitations. The study cohort is retrospective and contains a relatively small number of patients, with incomplete hospital data. We have limited information on the clinical decision-making that triggered the individual HEMS teams to transfuse. Detailed hospital haematology data may also have aided quantifying the effect of pre-hospital transfusion on coagulopathy. A confounding factor in this study is that patients receiving PRBC were likely to be the most severely injured and therefore require more interventions and pre-hospital time. These patients are likely to have the highest degrees of coagulopathy and worse outcome. This study was unable to link with certainty pre-hospital transfusion to improved outcome but the sample size is relatively small. Further, larger studies are warranted to determine whether pre-hospital transfusion of packed red cells can improve outcome.

## Conclusion

Pre-hospital PRBC transfusion is possible with complete traceability. Pre-hospital PRBC transfusion significantly reduces the time to transfusion for major trauma patients with suspected major haemorrhage and improves physiology during the pre-hospital phase. Administration of PRBC was associated with improved return of spontaneous circulation rate following traumatic cardiac arrest. The majority of patients receiving pre-hospital PRBC were severely injured and required further transfusion in hospital. Further research is warranted to determine which patients are most likely to have outcome benefit from pre-hospital blood products and what triggers should be used for pre-hospital transfusion.

## Abbreviations

ED: Emergency Department
FFP: Fresh frozen plasma
HEMS: Helicopter emergency medical service
IQR: Inter quartile range
ISS: Injury severity score
ITU: Intensive therapy unit
KSSAAT: Kent Surrey & Sussex Air Ambulance Trust
MOI: Mechanism of injury
NAO: National audit office
PRBC: Packed red bloods cells
ROSC: Return of spontaneous circulation
RSI: Rapid sequence induction
RTC: Road traffic collision
SD: Standard deviation
SOP: Standard operating procedure
TCA: Traumatic cardiac arrest

## References

[1] Lockey D, Crewdson K, Davies G (2006). Traumatic cardiac arrest: who are the survivors?. Ann Emerg Med.

[2] Norfolk D. Handbook of Tranfusion Medicine. United Kingdom Blood Services. Norwich: TSO information & publishing solutions; 2013.

[3] Barbosa RR, Rowell SE, Sambasivan CN, Diggs BS, Spinella PC, Schreiber MA, Holcomb JB, Wade CE, Brasel KJ, Vercruysse G (2011). A predictive model for mortality in massively transfused trauma patients. J Trauma.

[4] Guidelines for transfusion for massive blood loss (1988). A publication of the British society for haematology. British committee for standardization in haematology blood transfusion task force. Clin Lab Haematol.

[5] Jansen JO, Thomas R, Loudon MA, Brooks A. Damage control resuscitation for patients with major trauma. BMJ. 2009;338(b1778). https://doi.org/10.1136/bmj.b1778. 10.1136/bmj.b1778 19502278

[6] Holcomb JB, Donathan DP, Cotton BA, Del Junco DJ, Brown G, Wenckstern TV, Podbielski JM, Camp EA, Hobbs R, Bai Y (2015). Prehospital transfusion of plasma and Red blood cells in trauma patients. Prehosp Emerg Care: off j Natl Assoc EMS Physicians Natl Assoc State EMS Dir.

[7] Doughty HA, Woolley T, Thomas GO (2011). Massive transfusion. J R Army Med Corps.

[8] Ball CG, Salomone JP, Shaz B, Dente CJ, Tallah C, Anderson K, Rozycki GS, Feliciano DV (2011). Uncrossmatched blood transfusions for trauma patients in the emergency department: incidence, outcomes and recommendations. Can J Surg.

[9] Weaver A, Eshelby S, Norton J, Lockey D (2013). The introduction of on-scene blood transfusion in a civilian physician-led pre-hospital trauma service. Scand J Trauma Resusc Emerg Med.

[10] Sherren P, Burns B (2013). Prehospital blood transfusion: 5-year experience of an Australian helicopter emergency medical service. Crit Care.

[11] Bodnar D, Rashford S, Hurn C, Quinn J, Parker L, Isoardi K, Williams S. Characteristics and outcomes of patients administered blood in the prehospital environment by a road based trauma response team. Emerg Med J. 2014;31(7):583–8. 10.1136/emermed-2013-202395 23645008

[12] Bodnar D, Rashford S, Williams S, Enraght-Moony E, Parker L, Clarke B (2014). The feasibility of civilian prehospital trauma teams carrying and administering packed red blood cells. Emerg Me J.

[13] Brown JB, Sperry JL, Fombona A, Billiar TR, Peitzman AB, Guyette FX. Pre-Trauma Center Red Blood Cell Transfusion Is Associated with Improved Early Outcomes in Air Medical Trauma Patients. J Am Coll Surg. 2015;220(5):797–808. 10.1016/j.jamcollsurg.2015.01.006 PMC4409495 25840537

[14] Willis CD, Cameron PA, Bernard SA, Fitzgerald M (2006). Cardiopulmonary resuscitation after traumatic cardiac arrest is not always futile. Injury.

[15] Sihler KC, Napolitano LM (2010). Complications of massive transfusion. Chest.

[16] Denlinger JK, Nahrwold ML, Gibbs PS, Lecky JH (1976). Hypocalcaemia during rapid blood transfusion in anaesthetized man. Br J Anaesth.

[17] Fuenfer M, Creamer K (2013). Pediatric surgery and medicine for hostile environments.

[18] Jansen JO, Morrison JJ, Midwinter MJ, Doughty H (2014). Changes in blood transfusion practices in the UK role 3 medical treatment facility in Afghanistan, 2008–2011. Transfus medi (Oxford, England).

[19] Trust KSSAA. Marden, Kent, UK: KSS Air Ambulance; Kent, Surrey & Sussex Air Ambulance Trust. 2013. https://www.kssairambulance.org.uk.

[20] Major Trauma Care in England. National Audit Office. Report by the Comptroller and Auditor General. HC 213. Session 2009–2010. Published 5 February 2010.

[21] Joint United Kingdom (UK) Blood Transfusion and Tissue Transplantation Services Professional Advisory Committee. 2007 SI. 2007. http://www.transfusionguidelines.org.uk/regulations/archive/mhra-requirements.

[22] Trust KSSAA. Marden, Kent, UK: KSS Air Ambulance; Kent, Surrey & Sussex Air Ambulance Trust. 2014. https://www.kssairambulance.org.uk.

[23] Mackenzie CF, Wang Y, Hu PF, Chen SY, Chen HH, Hagegeorge G, Stansbury LG, Shackelford S (2014). Automated prediction of early blood transfusion and mortality in trauma patients. J Trauma Acute Care Surg.

[24] Lockey DJ, Lyon RM, Davies GE (2013). Development of a simple algorithm to guide the effective management of traumatic cardiac arrest. Resuscitation.

[25] Brockamp T, Nienaber U, Mutschler M, Wafaisade A, Peiniger S, Lefering R, Bouillon B, Maegele M (2012). Predicting on-going hemorrhage and transfusion requirement after severe trauma: a validation of six scoring systems and algorithms on the TraumaRegister DGU. Crit Care.

[26] Spinella PC, Holcomb JB (2009). Resuscitation and transfusion principles for traumatic hemorrhagic shock. Blood Rev.

[27] Dailey RH (1985). “Code Red” protocol for resuscitation of the exsanguinated patient. J Emerg Med.

[28] Metheny N (2011). Fluid and electrolyte balance.

[29] Vivien B, Langeron O, Morell E, Devilliers C, Carli PA, Coriat P, Riou B (2005). Early hypocalcemia in severe trauma. Crit Care Med.

